# Effects of modified sleeper stretch and modified cross-body stretch on upper limb functions and shoulder ROM in tennis players: a randomized trial

**DOI:** 10.1038/s41598-023-35977-w

**Published:** 2023-06-05

**Authors:** Anjupriya D., Aparna Sudhan M., Shilpa Chandran, Shibili Nuhmani, Mohammad Ahsan, Ahmad H. Alghadir, Masood Khan

**Affiliations:** 1 Co-operative Institute of Health Sciences, Thalassery, Kerala India; 2 Department of Physical Therapy, College of Applied Medical Sciences, Imam Abdulrahman Bin Faisal University, Dammam, Saudi Arabia; 3 Department of Rehabilitation Sciences, College of Applied Medical Sciences, King Saud University, P.O. Box. 10219, Riyadh, 11433 Saudi Arabia

**Keywords:** Rehabilitation, Muscle

## Abstract

Tennis players often experience posterior shoulder pain due to restricted internal rotation (IR) range of motion (ROM) of the glenohumeral joint. No research has compared the effects of modified sleeper stretch (MSS) versus modified cross-body stretch (MCBS) on tennis players’ upper limb functions and IR ROM. The study aimed to compare the efficacy of modified sleeper and cross-body adduction stretch in improving shoulder IR ROM and upper limb functions in tennis players. Thirty male lawn tennis players (aged 20 to 35 years) with more than 15° glenohumeral IR deficiency on the dominant side compared to the non-dominant side were recruited and divided into two groups: Modified sleeper stretch group (MSSG) and modified cross-body stretch group (MCBSG). MSSG received MSS, and MCBSG received MCBS, 3–5 repetitions once daily for 4 weeks. Upper limb functions were measured using the Disability of the Arm, Shoulder, and Hand (DASH) scale, and the IR ROM of the shoulder joint was measured using a universal goniometer. Both groups observed significant (p < 0.05) DASH scores and IR ROM improvements. DASH scores decreased by 85% in MSSG and 79.60% in MCBSG. IR ROM increased by 94.64% in MSSG and 89.52% in MCBSG. No significant differences (p > 0.05) were found in post-intervention DASH scores and IR ROM values between both groups. MSS and MCBS improved upper limb functions and IR ROM of the shoulder joint in the selected sample population of lawn tennis players. No difference was observed between both stretching techniques in improving upper limb functions and IR ROM of the shoulder joint.

## Introduction

Tennis players often experience posterior shoulder pain due to restricted internal rotation (IR) range of motion (ROM) at the shoulder joint. One of the most common causes of shoulder pain and limited IR ROM has been reported to be posterior shoulder stiffness or contracture^1^. A reduced ROM of the glenohumeral joint can affect shoulder performance^2^. Loss of IR at the glenohumeral joint is common in overhead athletes, known as glenohumeral internal rotation deficiency (GIRD)^3^. Tightness of the posterior soft tissues of the shoulder joint (posterior shoulder capsule, rotator cuff, and posterior deltoid muscle) may lead to impingement syndrome, rotator cuff injuries, or labral lesions^4^. Young, vigorous overhead athletes, particularly tennis players, are more likely to have internal impingement^5,6^.

Tennis games place high pressure on the players' joints, with supraphysiological forces generated hundreds of times per match in the shoulder and elbow joints. It has been reported that acute injuries frequently occur in the lower extremities among tennis players, whereas chronic injuries frequently occur in the upper extremities^7^. The tennis serve is a complicated stroke that involves segmental rotations throughout the kinetic chain. Repeated external rotations during the tennis serve’s cocking phase may reduce the ROM for IR and increase the ROM for external shoulder rotation in the dominant arm. The deceleration phase of the serve creates a large compressive force on the shoulder of the player^8^. These repetitive forces have been suggested to result in secondary changes in the muscles present posteriorly to the shoulder joint (teres minor, infraspinatus, posterior deltoid, and latissimus dorsi) and shoulder joint capsule that result in altered ROM^9,10^. Due to these changes, some scapular biomechanic adaptations favor biomechanical obstructions that increase the risk of rotator cuff musculotendinous injury in an athlete^11,12^. These adaptations include a decrease in the upward rotation of the scapula, an increase in sternoclavicular elevation, an increase in anterior tilt and protraction of the scapula, and a dropped scapula in the rest position^13,14^. Posterior shoulder stiffness can contribute to alteration in the rotational axis of the head of the humerus. This sort of stiffness causes the head of the humerus to shift in a superior and posterior direction, causing an increase in external rotation and a decrease in IR ROM, ultimately resulting in an abnormality in the shoulder ROM of the athletes.

Overhead athletes frequently perform posterior shoulder stretching as part of prevention programs to reduce the risk of shoulder injuries^15^. The sleeper stretch was described by Burkhart et al.^16^ in which the athlete's shoulder and elbow of the side to be stretched are flexed to 90° while lying in the side-lying position on the throwing side to maintain the scapula over the table. In this position, passive IR is performed on the ipsilateral side by the hand of the contralateral side^16^. Horizontal adduction stretch or cross-body stretch is reported to improve IR of the shoulder joint^17^. There is an inability during the cross-body stretch to control the rotation at the glenohumeral joint and stabilize the scapula, and there is a tendency to cause subacromial impingement during sleeper’s stretch; therefore, several adjustments are advised for both of these frequently used stretches. Wilk et al.^18^ proposed a modified sleeper stretch (MSS), in which the athlete will be in a side-lying position with the trunk rolled 20° to 30° posteriorly, and the shoulder elevated 90°. The athlete will then perform the passive IR with his/her opposite arm. The MSS is intended to reduce the possibility of shoulder pain when the shoulder is flexed to 90°. In this posture, the humerus is oriented in the scapular plane, putting more strain on the posterior capsule (scapular plane). A modified cross-body stretch (MCBS) in a side-lying position is performed to stabilize the scapula against the table and abduct the shoulder horizontally^18^.

Yamauchi et al.^19^ have compared the effects of MSS and MCBS in baseball players. They examined the effects of these stretching techniques on ROM and muscle stiffness. No studies have compared the effects of these stretching techniques on upper limb functions and IR ROM in tennis players. Therefore, the present study was conceptualized to examine and compare the effects of MSS and MCBS on upper limb functions and IR ROM of the shoulder joint in lawn tennis players. The present study also aimed to find the best technique from these two to improve upper limb functions and IR ROM. We hypothesized that there is a significant difference between the effects of these stretching techniques on upper limb functions and IR ROM.

## Materials and methods

### The experimental approach to the problem

A parallel group design was used to determine whether an MSS and an MCBS improve the upper limb functions and shoulder joint's IR ROM. Upper limb functions and IR ROM were the dependent variables, and the two stretching techniques were the independent variables.

### Participants, randomization, and ethical approval

**Figure 1:**
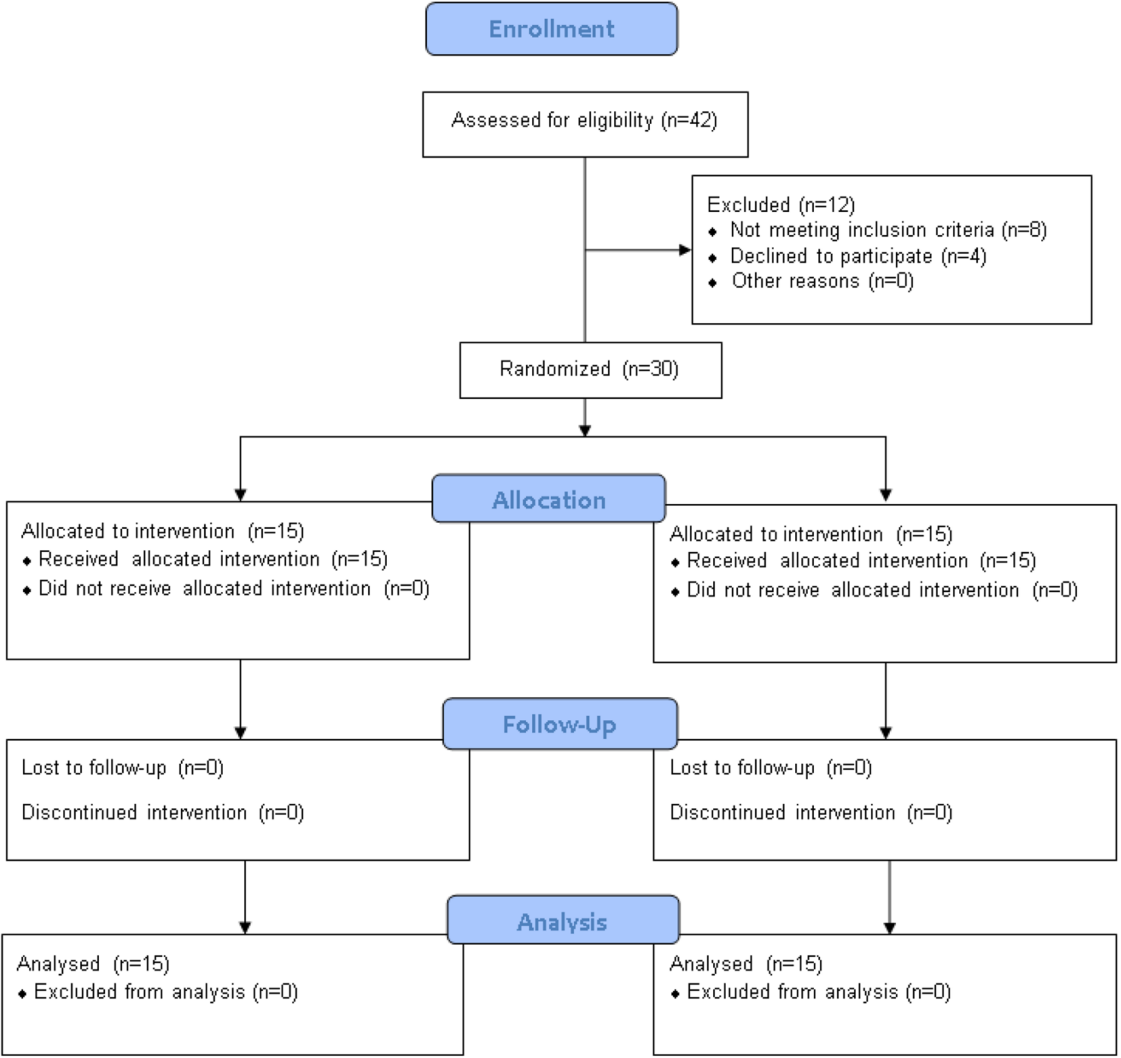
Consolidated Standards of Reporting Trials (CONSORT) flow chart showing the number of assessed, recruited, and analyzed participants in both groups.

A minimum group size of 30 individuals is deemed necessary for experimental research to make a valid generalization^20,21^. Therefore, thirty male lawn tennis players aged between 20 and 35 years, with more than 15° GIRD on the dominant shoulder (compared to the non-dominant shoulder), were recruited for the study (Fig. 1). The anthropometric characteristics of the participants are presented in Table 1. Participants with systemic or metabolic disorders, a positive test for labral lesions or rotator cuff tears, a history of recent fracture, or orthopedic surgery in the upper limbs or cervical region were excluded from the study. Participants were instructed to continue with their regular diet and training activities.

**Table 1:** Demographic characteristics, variables data, and p-values for the Shapiro–Wilk test of normality.

	MSSG (n = 15)		MCBSG (n = 15)	
	Mean ± SD	p-value	Mean ± SD	p-value
Age (years)	25.40 ± 3.71		26.26 ± 3.41	
Height (cm)	167.00 ± 6.17		169.73 ± 7.18	
Weight (kg)	68.40 ± 8.87		67.46 ± 10.74	
IR_ROM_Pre (degree)	35.86 ± 8.08	0.161	36.86 ± 6.74	0.107
IR_ROM_Post (degree)	69.80 ± 7.27		69.86 ± 6.47	
DASH_Pre (points)	25.00 ± 19.90	0.001*	22.46 ± 12.46	0.004*
DASH_Post (points)	3.75 ± 5.68		4.58 ± 7.99	

MSSG: modified sleeper stretch group; MCBSG: modified cross-body stretch group; SD: standard deviation; IR: internal rotation; ROM: range of motion; DASH: disability of the arm, shoulder, and hand.

*Significant.

A familiarization session was held before the intervention to ensure the participants felt at ease with the study protocol. Participants were randomly allocated into the Modified sleeper stretch group (MSSG) and the Modified cross-body stretch group (MCBSG) by a physical therapist. This physical therapist was not associated with the study. For randomization, the lottery method and the website randomization.com was used. Written informed consent was obtained from each participant before the study initiation, and the Code of Ethics of the World Medical Association, the Declaration of Helsinki were followed. The institutional ethics committee of the Co-Operative Institute of Health Sciences, Thalassery, Kannur (IRB No: 3/2015/MPT-Musculoskeletal & Sports/CIHS) approved the study. The study was conducted at Trivandrum Tennis Club, Thiruvananthapuram, and Lakshmibai National College of Physical Education. The study has been registered on clinicaltrials.gov (Protocol Registration and Results System, ID: NCT05540301, date: 14/09/2022). The outcome measures were assessed by another physical therapist blinded to the participants' allocation.

### Outcome measures

#### Upper limb functions

Upper limb functions of the dominant side were measured using the Disability of the Arm, Shoulder, and Hand (DASH) scale. DASH scale is a self-administered region-specific outcome scale consisting of 30-item, with each module consisting of 4 items. It measures the physical function and symptoms of people with musculoskeletal diseases in the upper limbs^22^.

#### Internal rotation range of motion

ROM was measured using the universal goniometer^23^. The reliability and validity of the universal goniometer in measuring shoulder internal rotation ROM have been well-established in previous studies^24,25^. The measurements were taken by an expert physical therapist with more than 7 years of clinical experience, who was blinded to the allocation of the participants. The participants were made to lie in the supine lying position, and the plinth supported the tested arm. The shoulder joint was abducted 90°, the elbow flexed 90°, and the wrist was neutral. A small towel roll was placed under the arm for stabilization. Then the participants were instructed to internally rotate the arm while maintaining the shoulder in the abducted position. The measurements were taken with the stationary arm of the goniometer parallel to the floor, the movable arm parallel to the forearm and the fulcrum at the olecranon process^26,27^. A total of three readings were taken and the average of these three was used for the data analysis.

### Study procedure

Baseline measurements of the DASH scale and IR ROM were taken 24 h before the start of the 4-week stretching protocol, and the post-test measurements were performed 24 h after the end of the stretching protocol. MSSG received a modified sleeper stretch (MSS), and MCBSG received a modified cross-body stretch (MCBS) for 4 weeks, 3–5 repetitions once daily. Stretching was performed in the morning (7–9 a.m.), and participants were asked not to stretch and play the game on the day of baseline and post-intervention evaluation.

### Modified sleeper stretch (MSS)

The participants were made to lie side-lying with the dominant arm downward, the trunk rotated posteriorly 20 to 30°, the shoulder raised to 90°, the elbow flexed to 90°, and both knees semi-flexed to ensure stability. Participants were asked to use the opposite hand to grasp the dominant hand below the wrist and gradually internally rotate the forearm towards the couch; i.e., the participant performed passive IR with the opposite arm^18^. The physical therapist held the participants’ backs to avoid further trunk rotation. This stretch position was to be maintained for 30 s and performed with 3–5 repetitions once daily for 4 weeks.

### Modified cross-body stretch (MCBS)

The participants were side-lying on the dominant side, with the trunk rotated posteriorly 20° to 30°, the shoulder raised to 90°, the elbow flexed to 90°, and both legs semi-flexed. The physical therapist avoided further trunk rotation by holding the participant's back. The participants grasped the distal end of the humerus of the dominant side with the other hand and kept the forearm of the non-dominant side on top of the forearm of the dominant side. Thereby limiting the external rotation of the dominant side with the opposite forearm's counter-pressure. Then, participants need to horizontally adduct the humerus of the dominant side across their body with the help of their opposite hand^18^. This stretch position was to be maintained for 30 s and performed with 3–5 repetitions once daily for 4 weeks.

### Statistical analysis

IBM SPSS statistical software version 26 (SPSS Inc., Chicago, IL, USA) for Windows was used for the statistical analysis. Means and standard deviations (SD) were used to describe the data. The Shapiro–Wilk test of normality was used to assess the normal distribution of the baseline values of the dependent variables (DASH scores and IR ROM). This test revealed a normal distribution of the baseline values of IR ROM and no normal distribution of DASH scale scores in both groups. Therefore, non-parametric tests were used for further with-in (Wilcoxon signed-rank test) and between-group (Mann–Whitney U test) analyses. This study considered the type-I error of less than 0.05 acceptable. The effect size was also calculated using the formula r = z/√n.

### Informed consent

Written informed consent was obtained from all participants prior to the start of the study.

## Result

Data from 30 participants were analyzed, with 15 participants in each group.

*With-in group analysis:* Table 2 depicts the with-in group results.

**Table 2:** With-in group (Wilcoxon signed-rank test) results for both groups.

	MSSG			MCBSG		
	Z	p-value	Effect size	z	p-value	Effect size
IR_ROM_Post–IR_ROM_Pre	− 3.410	0.001*	− 0.622	− 3.408	0.001*	− 0.622
DASH_Post–DASH_Pre	− 3.425	0.001*	− 0.625	− 3.436	0.001*	− 0.627

MSSG: modified sleeper stretch group; MCBSG: modified cross-body stretch group; IR: internal rotation; ROM: range of motion; DASH: disability of the arm, shoulder, and hand.

*Significant.

A significant reduction (p = 0.001) in DASH scale scores was observed in both groups. DASH scale scores decreased by 85% in MSSG (effect size = − 0.625) and 79.60% in MCBSG (effect size = − 0.627).

A significant increase (p = 0.001) in IR ROM was observed in both groups. IR ROM increased by 94.64% in MSSG (effect size = − 0.622) and 89.52% in MCBSG (effect size = − 0.622).

*Between-group analysis:* Table 3 depicts the between-group results.

**Table 3:** Between-group (Mann–Whitney U test) results.

	Z	p-value	Effect size
IR_ROM_Post	− 0.063	0.950	− 0.011
DASH_Post	− 0.121	0.904	− 0.022

IR: internal rotation; ROM: range of motion; DASH: disability of the arm, shoulder, and hand.

No significant differences (p > 0.05) were found in the post-intervention DASH scale scores (effect size = − 0.022) and IR ROM (effect size = − 0.011) values between both groups.

## Discussion

The results of the present study show significant improvements in both groups' DASH scores and IR ROM. However, no significant differences were observed when both groups were compared, indicating that both stretchings were equally effective in improving DASH scores and IR ROM. Therefore, any of these two stretchings can be used for these purposes.

Posterior shoulder contracture is a common cause of shoulder pain in people with restricted IR or glenohumeral internal rotation deficiency (GIRD)^1^. Many studies have indicated overhead athletes had more external rotation and less IR at shoulder joints. Marcondes et al.^11^ studied 49 amateur tennis players to measure posterior shoulder stiffness, rotator cuff strength, and posterior shoulder tightness. The authors reported severe posterior capsule tightness and reduced IR ROM in athletes with shoulder pain. According to Manske et al.^3^, the overhead athlete will not have the required external rotation to serve a tennis ball at 120 miles per hour or more velocities without losing the IR at the glenohumeral joint. Myers et al.^2^ reported a relationship between posterior shoulder tightness and impingement syndrome. Harryman et al.^28^ demonstrated that during passive shoulder flexion, selective tightness of the posterior region of the shoulder capsule generates an obligatory anterior and superior translation of the humeral head. This irregular motion could cause a soft tissue impingement in the subacromial region in those requiring overhead sports or work activities.

The two stretching interventions used in the present study, MSS and MCBS, stretched the posterior soft tissues of the shoulder joint, thereby increasing the glenohumeral IR ROM and decreasing the posterior shoulder stiffness. IR ROM increased by 94.64% in MSSG and 89.52% in MCBSG. Although greater improvement was observed in MSSG than in MCBSG, this difference was not statistically significant. A similarly greater difference was observed in DASH scores in MSSG than in MCBSG (decreased by 85% in MSSG and 79.60% in MCBSG); this difference was also not statistically significant. The non-statistically significant difference between both groups for both outcome measures could be due to the present study's small sample size (n = 30). A large sample size may reveal a statistically significant difference between MSSG and MCBSG.

Overhead athletes commonly perform posterior shoulder stretching as a part of the shoulder injury prevention program. Burkhart et al.^29^ described that sleeper stretch is performed with the person side-lying on the affected side with the shoulder and elbow flexed to 90°; the non-affected hand performs passive IR of the affected side. This position may result in subacromial impingement. Burkhart et al.^30^ also described the rollover sleeper stretch where the shoulder is only flexed 50° to 60°, and the person is rolled forward 30° to 40° from vertical. The authors believed the rollover sleeper stretch is a forceful method that might cause pain in many athletes and should be performed cautiously.

Tyler et al.^17^ described a cross-body or horizontal adduction stretch to improve shoulder IR where the affected arm is lifted to around 90° flexion. Then they pushed across the body into horizontal adduction with the force of the opposite arm. The inadequacy of this stretching method to selectively stretch the posterior capsule has been challenged. Although no biomechanical studies or tissue strain testing have been conducted to approve or disapprove this theory, physicians believe that the scapulothoracic tissues may be stretched^3^. This stretch can be considered if it is necessary to stretch the posterior soft tissues (rather than the posterior capsule). Wilk et al.^18^ reported a stretching technique called horizontal adduction stretch with scapular stabilization that selectively stretches the glenohumeral joint's posterior tissues. The subject is supine as the therapist stabilizes the scapula with one hand and applies a horizontal adduction moment to the humerus with the other hand. Because the therapist stabilizes the scapula, the posterior scapular muscle should get less tissue stretch.

Salamh et al.^31^ showed that horizontal adduction stretch performed with scapular stabilization improved IR ROM and posterior shoulder tightness more than horizontal adduction stretch without scapular stabilization. Wilk et al.^18^ proposed modifications for these two commonly performed stretches, MSS and MCBS, which are the keystone of this study. McClure et al.^32^ compared the various stretching procedures used to reduce posterior shoulder tightness and showed that cross-body stretch is more effective than sleeper stretch. These authors suggested that it may be due to pain or the inconvenient position during the sleeper stretch position. Our study showed that pain and discomfort were significantly less with MSS. Another study by Laudner et al.^9^ showed that sleeper stretch produces a statistically significant acute improvement in posterior shoulder tightness. In MCBS the scapula is stabilized better than the conventional cross-body stretch, thereby increasing shoulder IR ROM more effectively.

Several limitations and scopes for future research should be mentioned, like that the ROM measurements were performed manually, leading to the possibility of human error and reducing the study's accuracy. The participants selected in the present study were without pain or symptoms. The study should be carried out on participants with pain and other symptoms and a large sample size to establish greater generalizability. Future studies should also be carried out in different overhead sports, and long-term follow-up is needed.

## Conclusion

MSS and MCBS improved upper limb functions and IR ROM of the shoulder joint in the selected sample population of lawn tennis players. No difference was observed between both stretching techniques in improving upper limb functions and IR ROM of the shoulder joint. Therefore, either of the two stretching techniques can be used to improve upper limb functions and IR ROM in those tennis players with more than 15° GIRD.

## Data Availability

The de-identified dataset used and/or analyzed during the current study are available from the first author on reasonable request.
